# Diagnostic accuracy of the peripheral venous pressure variation induced by an alveolar recruitment maneuver to predict fluid responsiveness during high-risk abdominal surgery

**DOI:** 10.1186/s12871-023-02194-x

**Published:** 2023-07-22

**Authors:** Olivier Desebbe, Sylvain Vallier, Laurent Gergelé, Brenton Alexander, Alexandre Marx, Elias Ben Jaoude, Hiromi Kato, Leila Toubal, Antoine Berna, Jacques Duranteau, Jean-Louis Vincent, Alexandre Joosten

**Affiliations:** 1 Department of Anesthesiology and Perioperative Medicine, Sauvegarde Clinic, Ramsay Sante, Lyon, France; 2 Department of Anesthesiology and Intensive Care, Elsan Alpes-Belledonne Clinic, Grenoble, France; 3 Department of Anesthesiology and Intensive Care, Ramsay Sante HPL Clinic, Saint-Etienne, France; 4 Department of Anesthesiology, University of California San Diego, La Jolla, CA USA; 5 Department of Anesthesiology, Erasme Hospital, Université Libre de Bruxelles, Brussels, Belgium; 6 Department of Anesthesiology and Intensive Care, Université Paris-Sud, Paul Brousse Hospital, Assistance Publique Hôpitaux de Paris (APHP), 12 Avenue Paul Vaillant Couturier, Villejuif, 94800 France; 7 Department of Intensive Care, Erasme Hospital, Université Libre de Bruxelles, Brussels, Belgium

**Keywords:** Alveolar recruitment maneuver, Fluid therapy, Cardio-pulmonary interactions, central venous pressure, Peripheral venous pressure, Hemodynamics, Mechanical ventilation

## Abstract

**Background:**

In patients undergoing high-risk surgery, it is recommended to titrate fluid administration using stroke volume or a dynamic variable of fluid responsiveness (FR). However, this strategy usually requires the use of a hemodynamic monitor and/or an arterial catheter. Recently, it has been shown that variations of central venous pressure (ΔCVP) during an alveolar recruitment maneuver (ARM) can predict FR and that there is a correlation between CVP and peripheral venous pressure (PVP). This prospective study tested the hypothesis that variations of PVP (ΔPVP) induced by an ARM could predict FR.

**Methods:**

We studied 60 consecutive patients scheduled for high-risk abdominal surgery, excluding those with preoperative cardiac arrhythmias or right ventricular dysfunction. All patients had a peripheral venous catheter, a central venous catheter and a radial arterial catheter linked to a pulse contour monitoring device. PVP was always measured via an 18-gauge catheter inserted at the antecubital fossa. Then an ARM consisting of a standardized gas insufflation to reach a plateau of 30 cmH_2_O for 30 s was performed before skin incision. Invasive mean arterial pressure (MAP), pulse pressure, heart rate, CVP, PVP, pulse pressure variation (PPV), and stroke volume index (SVI) were recorded before ARM (T1), at the end of ARM (T2), before volume expansion (T3), and one minute after volume expansion (T4). Receiver-operating curves (ROC) analysis with the corresponding grey zone approach were performed to assess the ability of ∆PVP (index test) to predict FR, defined as an ≥ 10% increase in SVI following the administration of a 4 ml/kg balanced crystalloid solution over 5 min.

**Results:**

∆PVP during ARM predicted FR with an area under the ROC curve of 0.76 (95%CI, 0.63 to 0.86). The optimal threshold determined by the Youden Index was a ∆PVP value of 5 mmHg (95%CI, 4 to 6) with a sensitivity of 66% (95%CI, 47 to 81) and a specificity of 82% (95%CI, 63 to 94). The AUC’s for predicting FR were not different between ΔPVP, ΔCVP, and PPV.

**Conclusion:**

During high-risk abdominal surgery, ∆PVP induced by an ARM can moderately predict FR. Nevertheless, other hemodynamic variables did not perform better.

**Supplementary Information:**

The online version contains supplementary material available at 10.1186/s12871-023-02194-x.

## Background

When caring for patients undergoing high-risk surgery, the judicious titration of fluid administration in order to avoid both hypo- and hypervolemia (and the resulting perioperative complications) is considered standard of care [1]. This is best achieved through well-informed and frequent assessments of each patient’s fluid responsiveness (FR) [2]. Static hemodynamic indicators such as mean arterial pressure (MAP) and central venous pressure (CVP) have been poorer indicators of FR than dynamic variables like pulse pressure variation (PPV) or stroke volume variation (SVV) [3]. Like PPV, CVP variations throughout the mechanical respiratory cycle have been shown to predict FR [4, 5]. More recently, variations of CVP during an alveolar recruitment maneuver (ARM) have also shown promise in predicting FR [6]. Clearly, the insertions of a central venous catheter is not always desirable for every type of patient’s perioperative management. In this context, peripheral venous pressure (PVP) has demonstrated a consistent and high degree of agreement with CVP in the perioperative period in patients without significant cardiac dysfunction [7, 8]. As a peripheral venous catheter is present in the vast majority of surgical patients, PVP measurement only requires an additional pressure sensor. Additionally, PVP have proven useful in determining FR in critically ill patients [9] and may be an indicator of preload responsiveness during volume resuscitation from hemorrhage [10].

The primary objective of this study was to test the hypothesis that dynamic variations of PVP (∆PVP) during an ARM can predict FR in mechanically ventilated patients undergoing high-risk abdominal surgery. Secondary objectives were (1) to compare FR predictability between ΔPVP and other hemodynamic variables (SVI, PPV, ΔPVC) and (2) to analyze the relationship between CVP and PVP by a linear regression and a Bland-Altman analysis.

## Methods

We applied the 2015 STARD guidelines for performing and reporting the results of this study [11], which took place at Paul Brousse Hospital between January 2022 and April 2022. The study protocol was approved on 17/06/2021 by the ethics committee of St Etienne (IRBN902021/CHUSTE) and registered on clinical trials (NCT05131516) on 23/11/2021 prior to the first patient inclusion. Informed consent was obtained before surgery in all patients.

Inclusion criteria were adult patients (≥ 18 years old) scheduled for a high-risk abdominal surgery and equipped with a radial arterial catheter connected to a cardiac output hemodynamic monitoring device (standard practice in our institution). Non-inclusion criteria were the presence of cardiac arrhythmias or known right ventricular dysfunction.

### Anesthesia protocol

Anesthesia management was standardized in all patients during the study period. Upon arrival in the operating room, patients were moved onto a heated mattress. The following noninvasive monitors were attached: 5-lead electrocardiogram, noninvasive blood pressure, rectal temperature probe, frontal lobe electroencephalogram (with a target Bispectral index of 40 to 60), and depth of neuromuscular block monitoring. A Foley catheter was introduced. Vascular access consisted of two large-diameter peripheral venous catheters (one 16-gauge for drug administration and another 18-gauge inserted at the antecubital fossa and connected to the patient monitor via a pressure catheter). Care was taken to ensure that the patient’s arm with the peripheral intravenous catheter was unobstructed by draping, tucking, or positioning. A radial arterial catheter was also inserted after anesthesia induction and connected via the Flotrac sensor to an uncalibrated pulse contour analysis monitoring device (EV1000, Edwards Lifesciences, Irvine, USA). Then a 16 cm, 8.5 F triple-lumen central venous catheter (Arrow International Inc, Division of Teleflex Medical Inc, Everett, MA, USA) was placed under ultrasound guidance into the right jugular vein. All pressure transducers (Medex Medical Ltd, Rossendale, Lancashire, UK) were placed at the level of the mid-axillary line. General anesthesia was induced with sufentanil and propofol with neuromuscular blockade being initially achieved using succinylcholine (if potassium was in the normal range) and maintained intraoperatively with atracurium. Maintenance of anesthesia was achieved using a sufentanil infusion and inhaled sevoflurane. All patients received mechanical ventilation using a volume control mode with tidal volumes of 7 to 8 ml/kg of predicted body weight, a positive end expiratory pressure (PEEP) of 5 cm H_2_0, and a respiratory rate adjusted to achieve an end tidal carbon dioxide between 35 and 40 cm H_2_O. Baseline fluid administration consisted of a balanced crystalloid infusion (Ringer’s lactate) at a rate of 1 ml/kg/h and fluid challenges of 4 ml/kg to optimize SVI during surgery.

### Alveolar recruitment maneuver and data collection

**Fig. 1:**
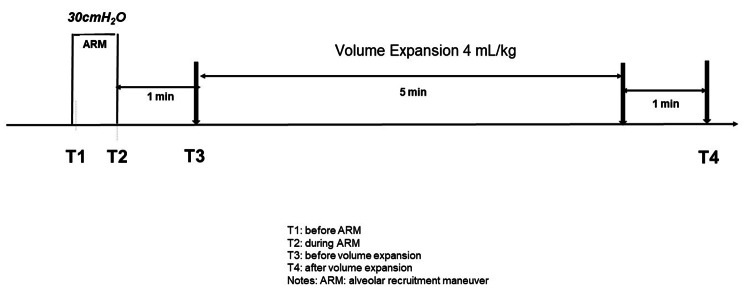
Study design. Notes: T1: before ARM; T2: during ARM; T3: before volume expansion; T4: after volume expansion. Legends: ARM: alveolar recruitment maneuver

An ARM was performed before skin incision, using an insufflation pressure of 30 cmH_2_O for 30 s [12]. Invasive systolic arterial pressure (SAP), diastolic arterial pressure (DAP), systemic pulse pressure (SAP-DAP), MAP, heart rate (HR), CVP, PVP, PPV and SVI were recorded before the ARM (T1), at the end of the ARM (T2), before volume expansion (T3), and 1 min after a volume expansion of 4 mL/kg of balanced crystalloid solution (Ringer’s lactate) administered over 5 min (T4) (Fig. 1) [13]. After the ARM, ventilatory settings were set back to initial settings. The ARM was stopped if severe arterial hypotension (systolic arterial pressure less than 70 mmHg) or severe hypoxemia (SpO2 < 80%) developed [14]. Patients were classified as fluid responders if SVI increased by ≥ 10% following the volume expansion [15].

A video of the hemodynamic monitors was recorded during the study protocol, with the clinician announcing at each time points the different values. Hemodynamic values were later documented by pausing on the video during the two last seconds of each time plot.

### Statistics

All hemodynamic variables are presented as either mean (SD) or median (25th.

to 75th percentiles), depending on the normality of distribution of data (Kolmogorov–Smirnov test). Subjects were allocated according to the percentage change in SVI induced by volume expansion. Volume responders were defined by an increase of SVI ≥ 10% following the volume expansion.

We calculated the absolute values of the variations (∆) of the hemodynamic parameters between the value at the plateau pressure of 30 cmH_2_0 (T2) and the value at the respiratory baseline (T1) for the following variables: ∆PVP, ∆CVP, ∆MAP, pulse pressure [(SAP-DAP); ∆PP]. We also recorded the respiratory variations of the arterial pulse pressure induced by mechanical ventilation [pulse pressure variation (PPV)] at T1, T3, and T4.

In order to verify the reliability of SV throughout the study protocol, the correlation between SV measurements at the two baseline steps (T1 before ARM and T3 before volume expansion) was compared using a random-effects model to ensure that large changes did not occur [16].

Differences between T1 and T2, and between T3 and T4 were assessed by a Wilcoxon test or a paired sample t-test according to the distribution of the differences. Responder and non-responder group data were compared using a Mann-Whitney test. The relationship between PVP and CVP was determined by a linear regression analysis plotting all the paired data using the coefficient of determination R² and by a Bland-Altman analysis with multiple observations per individual. To assess the ability of measured/calculated parameters to identify responders of intravascular fluid administration, receiver-operating characteristic (ROC) curves were generated, varying the discriminating threshold for ∆SVI, ∆CVP, ∆PVP, ∆MAP, ∆PP, and PPV. The optimal threshold value using the Youden index (the value that maximizes the sum of the sensitivity and specificity) was also determined. The areas under the ROC curves were calculated for each variable and compared as described previously [17]. We defined the grey zone for which strict conclusions could not be obtained as threshold values with a sensitivity lower than 90% or specificity lower than 90% [18].

A sample size of 55 patients was calculated to be sufficient to demonstrate that PVP variations could predict fluid responsiveness with an area under curve (AUC) above 0.75 [19], a ratio of non FR /FR groups of 1;1, with a power of 99% (beta risk = 0.01), an alpha risk of 0.01, and a null hypothesis value of 0.5. Considering a drop off around 10%, we decided to include 60 patients. Statistical analysis and sample size calculation were performed using MedCalc^®^ Statistical Software version 19.6.4 (MedCalc Software Ltd, Ostend, Belgium).

## Results

Sixty patients were included in the statistical analysis. No patients were excluded, and no patient was given any vasopressor medication. No patients experienced hemodynamic instability during the ARM requiring a cessation of the maneuver. Table 1 describes patient’s characteristics. Among the study collective, 32 patients (53%) were fluid responders and 28 (47%) were not. ICC for SVI between T1 and T3 was 0.98 (95% CI 0.97–0.99).

**Table 1:** Patient’characteristics

Variables	N = 60
Age	60 [45–72]
Sex, male N (%)	28 (47%)
Height (cm)	169 [162–181]
Weight (kg)	72 [60–84]
Tidal volume (ml/kg)*	7.6 [6.5–8.7]
ASA physical status II/III	22/38
Comorbidities (%)	
Arterial hypertension	65
Dyslipidemia	28
Diabetes	25
Ischemic cardiomyopathy	15
***Types of Surgery, n***	
Major hepatectomy	34
Duodenopancreatectomy	7
Biliary reconstruction	4
Liver transplantation	3
Exploratory laparotomy	12

*ideal body weight; ASA: american society of anesthesiologists

**Fig. 2:**
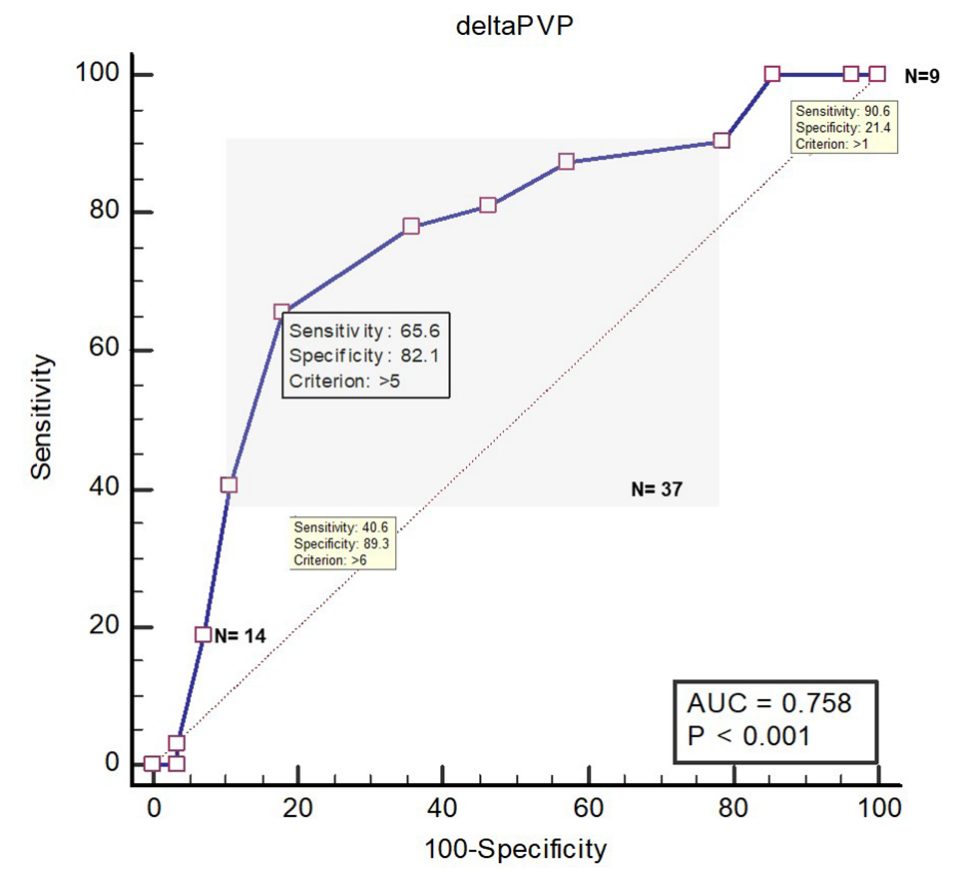
Receiver operating curves generated for changes in stroke volume index (SVI) induced by alveolar recruitment maneuver (ARM), changes in peripheral venous pressure induced by ARM, showing the ability to predict the effect of a volume expansion. Notes: alveolar recruitment maneuver consisted of applying a continuous positive airway pressure of 30 cm H_2_O for 30 s. The grey zone shows is delimited by two cut off values corresponding to a sensitivity of 90% (upper right corner) and a specificity of 90% (lower left corner). Values lying in the grey zone express a degree of uncertainty wherein the physician should pursue a diagnosis using additional tools. Legend: AUC: Area Under the Curve

Hemodynamic variables at the study baseline, during the ARM, before the fluid challenge and after the fluid challenge between responders and non-responders are shown in Table 2. ∆PVP during an ARM predicts FR with an area under the ROC curves of 0.76 (95% CI, 0.63 to 0.86). The optimal threshold was a ∆PVP value of 5 mmHg (95% CI, 4 to 6) with a sensitivity of 66% (95% CI, 47 to 81) and a specificity of 82% (95% CI, 63 to 94) (Fig. 2). Proportions of values lying in the grey zone (value presenting a sensitivity lower than 90% or a specificity lower than 90%) ranged from 43 to 67% according to the tested variable (Table 2). The AUC’s for predicting FR were not different between ΔPVP and other hemodynamic variables such as ΔCVP and PPV (Table 2; **Supplemental Fig. 1**).

**Table 2:** Hemodynamic Variables at Baseline, during Alveolar Recruitment Maneuver, before Volume Expansion, and after Volume Expansion in Responders (n = 32) and Non-responders (n = 28)

	T1 Before ARM	T2 During ARM	P1 value	T3 Before VE	T4 After VE	P2 value
PVP (mmHg)						
Responders	8 (6–12)	14 (12–18)	< 0.0001	9 ± 4	10 ± 4	0.0021
Non-responders	11 ± 5	15 ± 5	< 0.0001	11 (10–13)	11 (10–14)	0.7819
CVP (mmHg)						
Responders	8 (5–10)	13 (11–16)	< 0.0001	8 ± 3	9 ± 3	0.0026
Non-responders	9 ± 3	13 ± 4	< 0.0001	9 (6–12)	10 (9–12)	0.1650
MAP (mmHg)						
Responders	84 ± 11	78 ± 13	0.0001	85 ± 12	88 ± 16	0.3538
Non-responders	83 ± 9	81 ± 10	0.2192	83 ± 9	81 ± 10	0.2192
Pulse Pressure						
Responders	61 ± 14	48 ± 15	< 0.0001	61 ± 17	68 ± 15	0.0087
Non-responders	71 (52–81)	64 (48–74)	0.0004	65 ± 17	63 ± 17	0.3027
PPV (%)						
Responders	8 (6–11)	NA		9 (7–12)	6 (4–8)	0.0001
Non-responders	6 (4–8)	NA		6 (4–9)	5 (4–9)	0.2301
SVI (ml/min/m²)						
Responders	35 (31–41)	31 (25–38)	0.0001	32 (30–41)	38 (36–47)	< 0.0001
Non-responders	45 (37–48)	42 (35–45)	0.0001	44 (36–47)	45 (37–50)	0.0097

Notes:

Due to a short 30 s ARM period and a long averaging SVI values (20 s) from the pulse contour analysis, values of SVI during T2 should be interpreted with caution

Dispersion of the values is expressed in standard deviation (±) or in interquartile range (brackets)

Legends: ARM = Alveolar recruitment maneuver; NA: not applicable; P1 = comparison between T1 and T2; P2 = comparison between T3 and T4;

PPV = pulse pressure variations; pulse pressure = SAP-DAP; SVI: stroke volume index

T1 = baseline, before LRM; T2 = during LRM; T3 = baseline 2, before volume expansion; T4 = after 4 ml/kg crystalloid infusion; VE = volume expansion

**Fig. 3:**
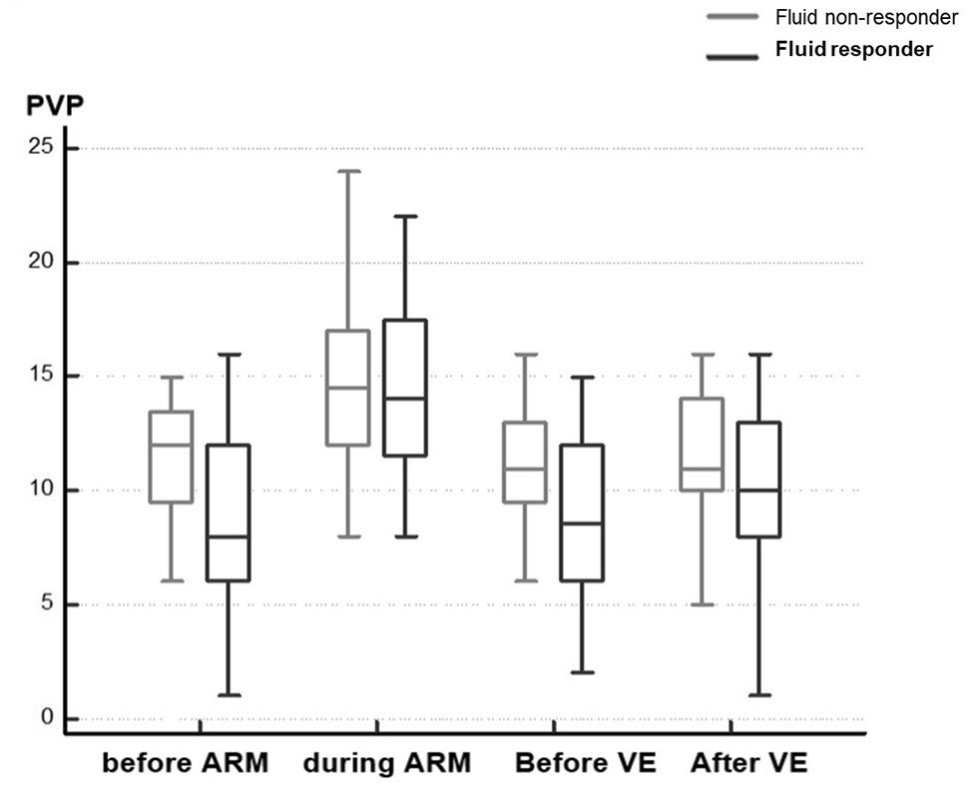
Evolution of the peripheral venous pressure at the 4 different time points between fluid responder patients and fluid non responder patients. Legends: ARM: Alveolar Recruitment maneuver; PVP: Peripheral Venous Pressure; VE: Volume expansion

PVP before the ARM in fluid responder patients was significantly lower than in non-responder patients (p = 0.0125). PVP increased significantly whatever the categorization of the patient (responder or not responder). Figure 3 depicts the evolution of PVP in responders and non-responders during the 4 time points. Table 3 shows the diagnostic performance of all hemodynamic variables to predict fluid responsiveness during the ARM.

**Table 3:** Diagnostic performance of hemodynamic variables to predict fluid responsiveness during ARM

Variables	AUC	95% CI	Cut-off	P value	grey zone			sensitivity	specificity	PPV	NPV
					**Lower**	**Upper**	**Patients** **n (%)**	(%)	(%)	(%)	(%)
**ΔPVP** (mmHg)	0.76	0.63 to 0.86	> 5	NA	1.2	6.2	37 (62)	66	82	81	68
**ΔCVP** (mmHg)	0.76	0.64 to 0.86	> 4	0.90	1.1	5.7	26 (43)	75	79	80	74
**ΔPP** (mmHg)	0.72	0.56 to 0.81	> 9	0.64	-1	18	35 (58)	66	79	78	67
**ΔMAP** (mmHg)	0.62	0.49 to 0.75	> 4	0.12	-5	13	46 (77)	63	68	69	62
**PPV** (%)	0.72	0.59 to 0.83	> 7	0.66	4	8	30 (50)	63	75	74	64

Legend: PPV: pulse pressure variations induced by the respiratory mechanical ventilation displayed by the scope; ΔCVP: difference between CVP before and CVP during ARM; ΔMAP : difference between MAP before and during ARM ; ΔPP : difference between pulse pressure (SAP-DAP) before and pulse pressure during ARM ; ΔPVP : difference between PVP before and PVP during ARM; NPV: negative predictive value; PPV: positive predictive value

The P value represents the pairwise comparison of ROC curves between the ΔPVP ROC curve and each tested ROC curves

**Fig. 4:**
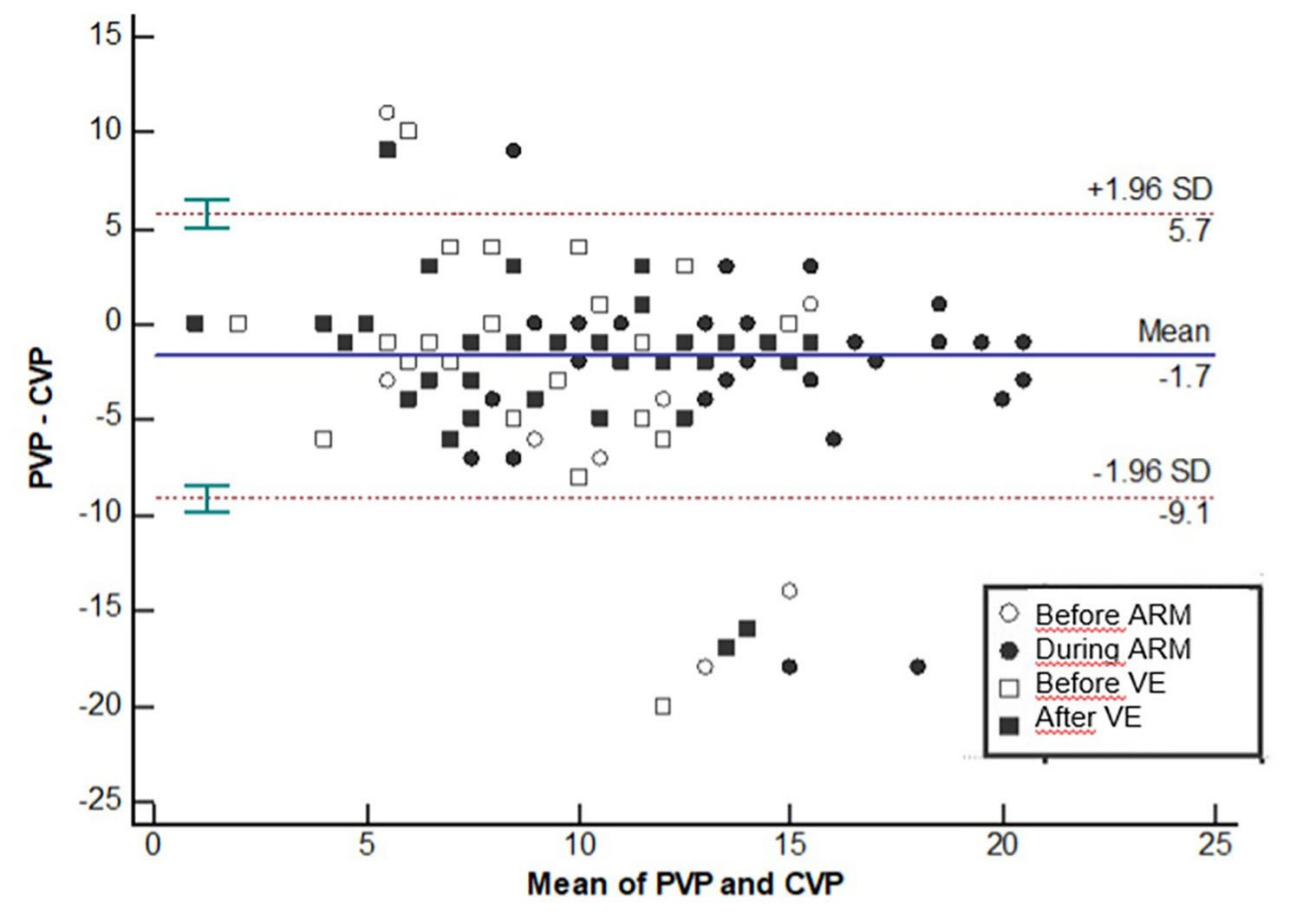
Bland–Altman plots showing the agreement between the PVP and the CVP with their limits of agreement (LOA) and the 95% CI of the LOA. Legends: ARM: Alveolar Recruitment maneuver; CVP: Central Venous Pressure; PVP: Peripheral Venous Pressure; VE: Volume expansion

The regression equation between PVP and CVP was significant (p < 0.0001; y = 4.44 + 0.71*x), with a coefficient of determination (R²) of 0.37 (**supplemental Fig. 2).** The Bland-Altman analysis with the limits of agreement and their 95% CI is presented Fig. 4.

## Discussion

The present study demonstrates that variation of the PVP induced by an ARM moderately predicted FR in mechanically ventilated patients in the operating room. It was not inferior to other commonly studied variables, including ΔCVP or PPV. To our knowledge, this is the first time that the variation of PVP induced by an ARM has been studied in surgical patients. However, despite a ROC curve value > 0.75 [19], and a relatively large number of patients compared to recent studies [13, 20, 21], a significant proportion of patient’s ∆PVP values were in the inconclusive grey zone. Indeed, ΔPVP may be useful only in 38% of the patients to assess fluid responsiveness. This highlights the difficulty and limitations of only using one hemodynamic variable to predict FR. A multimodal approach by combining other hemodynamic variables may limit the clinical uncertainty of FR.

Peripheral venous pressure is easy to measure and could be considered noninvasive as all surgical patients have at least one peripheral venous catheter. In this context, most patients may benefit from this easy and non-expensive monitoring wherein the insertion of an invasive arterial catheter or a CVP may not be necessary. Moreover, monitoring PVP during an ARM may be helpful when contraindications for the use of invasive PPV are present, such as atrial fibrillation, tidal volume < 8 mL/kg of ideal body weight, or a heart rate/respiratory rate ratio lower than 3.6 [22], especially as the percentage of patients without any contraindications has been documented between only 9% and 39% [23]. However, the potential added value of PPV to predict FR should be validated in future studies, including patients with a contraindication to the use of PPV.

PVP has been previously studied in a porcine model where it predicted FR during hypovolemic shock [10]. In cardiac surgery patients, Marques et al. found that PVP could not reliably predict FR, with an area under the ROC curve of 0.72 with large limits of agreement (95% confidence interval 0.52–0.92; p = 0.058) [24]. Importantly, the lack of dynamic tool to induce the PVP variation and the reduced left ventricular compliance may explain these results.

Our results showed a mild to moderate relationship between PVP and CVP (R²=0.37) with a Bland-Altman analysis showing large limits of agreement (+ 5.7/-9.1 mmHg). A Study observed an improved PVP to CVP correlation with increasing CVP values [25], another observed or a decrease of the PVP-CVP gradient after fluid loading [10] or during the ARM. Importantly, we measured PVP at the antecubital fossa and, as a result, did not measure the mean systemic filling pressure (msfP), which is the theoretical component of the driving pressure of the venous return (msfP-CVP) generated by the right heart [26]. PVP values may have been flawed by two conditions linked to the observational approach of this study. First, a collapse of the vein due to the absence of peripheral venous flow during low venous pressure states may have resulted in artificially low PVP values. Interestingly, the addition of a slow continuous flow of isotonic saline may prevent this collapsibility. Second, the contact between the venous catheter and endothelial wall may have resulted in artificially high PVP values. Performing an echographic assessment of the vein diameter and location of the distal position of the catheter may help decrease this inaccuracy. These two environmental constraints may partially explain the outliers of the Bland-Altman graph.”

The study has known limitations. First, the sample size calculation could have been further optimized by choosing a higher null hypothesis ROC curve value. We ultimately decided to use 0.5 as the null hypothesis AUC ROC curve value in order to avoid establishing an insurmountably large sample size requirement, at the known cost of a weakened statistical outcome. Secondly, our protocol was conducted prior to surgical incision, where hemodynamics are not affected by significant surgical-induced changes in sympathetic tone. However, the vasoplegia induced by the anesthesia induction may have affected pulse pressure by decreasing the DAP. Further studies are needed to explore if ΔPVP can predict FR during the intraoperative period, wherein fluid expansion is typically more complex. It is important to note that a fluid challenge is recommended before incision to optimize fluid status when stroke volume is measured [27]. Additionally, the initial apneic period following induction may have resulted in increased alveolar collapse and a potential exaggerated response to the ARM when compared to an intraoperative challenge. We also did not analyze the potential impact of the ARM on the venous waveform during abdominal insufflation or during different peak airway pressures [8]. Our study was oriented towards bedside applicability and the precise analysis of the venous waveform can be quite challenging in real time. Finally, our ARM lasted 30 s, wherein FloTrac values average hemodynamic values every 20 s. We therefore cannot accurately interpret ΔSVI during ARM during such a short period.

## Conclusion

In patients undergoing high-risk abdominal surgery, ∆PVP induced by an ARM can moderately predict FR with a high grey zone inclusion. Interestingly, other hemodynamic variables did not better predict FR. As the PVP values do not rely on additional expensive devices, the clinician may benefit from this additional hemodynamic variable to better optimize the patient’s fluid status. In the current era of enhanced recovery within the context of reducing health care costs, additional non-invasive and low-cost approaches may be warranted. However, further outcome studies based on these data should be performed to confirm its potential role in perioperative hemodynamic management.

## Electronic supplementary material

Below is the link to the electronic supplementary material.

Supplementary Material 1

Supplementary Material 2

Supplementary Material 3

Supplementary Material 4

## Data Availability

The database is closed and there is no public access. However, permission to access and use the database can be obtained if necessary by request to the corresponding author.

## List of abbreviations

ARM: Alveolar recruitment maneuver
AUC: Area under the curve
CVP: Central venous pressure
DAP: Diastolic Arterial Pressure
FR: Fluid Responsiveness
MAP: Mean Arterial PressurePVP:Peripheral venous pressure
PPV: Pulse Pressure Variation
ROC: receiver operating characteristics
SAP: Systolic Arterial Pressure
SVV: Stroke Volume Variation
SVI: Stroke Volume Index
