# Supplementary figures and images for: Diagnostic accuracy of the peripheral venous pressure variation induced by an alveolar recruitment maneuver to predict fluid responsiveness during high-risk abdominal surgery
Source: BMC Anesthesiol. 2023 Jul 22;23:249. doi: 10.1186/s12871-023-02194-x (PMC10362688; doi:10.1186/s12871-023-02194-x)

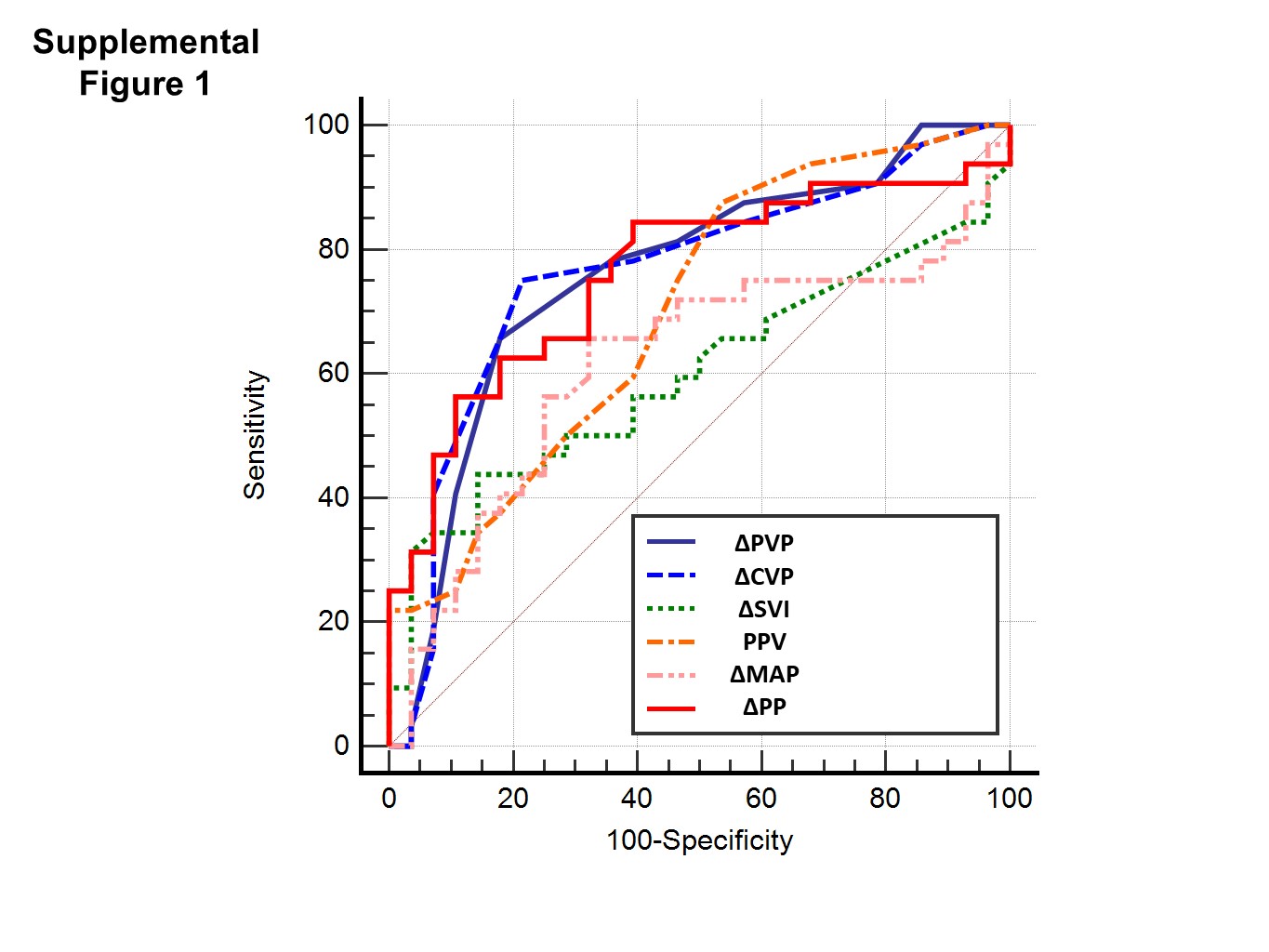

Supplement: Supplementary file 1 — Supplementary Material 1 [file 12871_2023_2194_MOESM1_ESM.jpg]

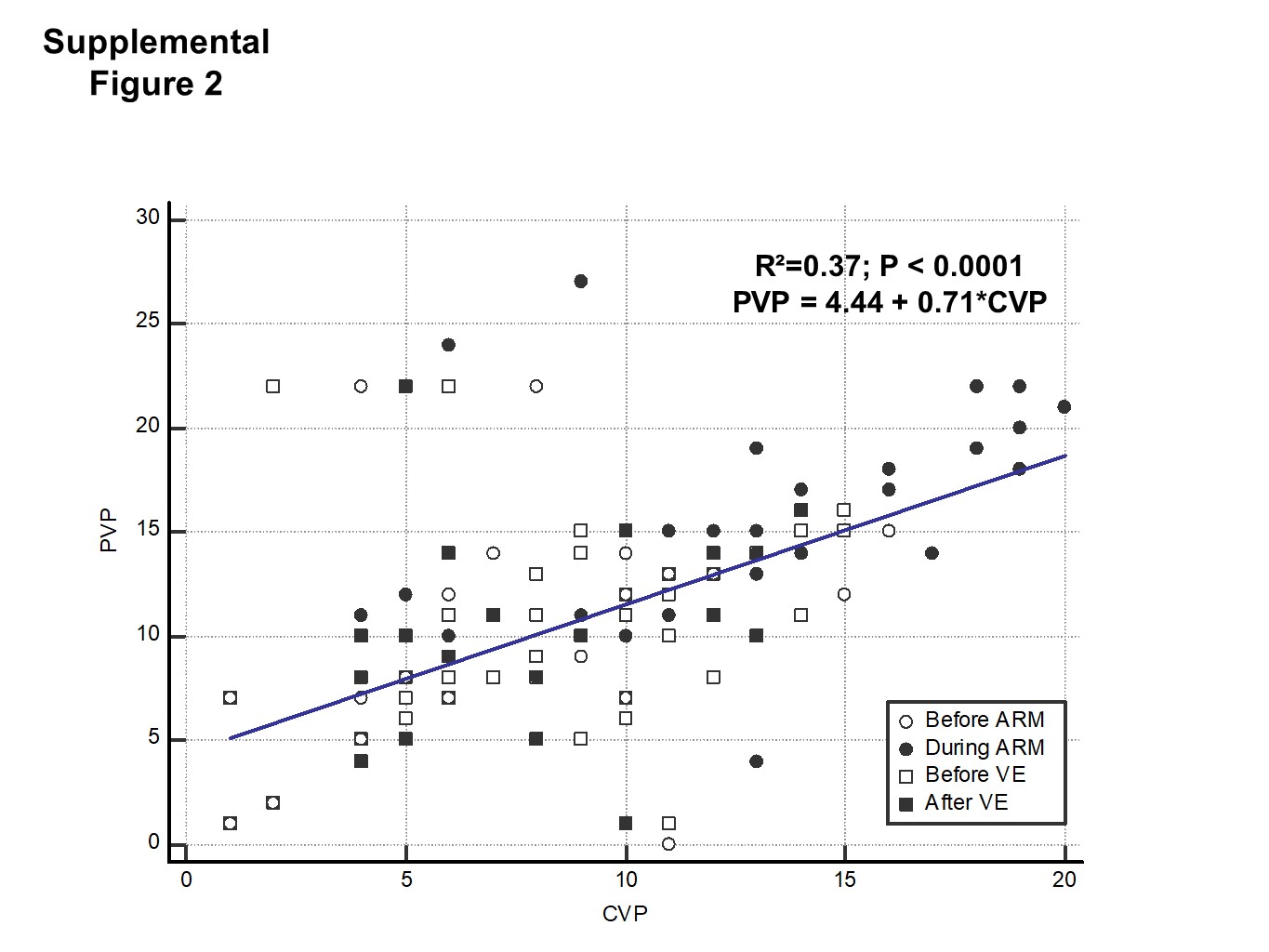

Supplement: Supplementary file 2 — Supplementary Material 2 [file 12871_2023_2194_MOESM2_ESM.jpg]
