# Supplementary material for: Diagnostic accuracy of the peripheral venous pressure variation induced by an alveolar recruitment maneuver to predict fluid responsiveness during high-risk abdominal surgery
Source: BMC Anesthesiol. 2023 Jul 22;23:249. doi: 10.1186/s12871-023-02194-x (PMC10362688; doi:10.1186/s12871-023-02194-x)
Supplement: Supplementary file 3 — Supplementary Material 3 [file 12871_2023_2194_MOESM3_ESM.docx]

**Supplementary Figure 2**:

Bland–Altman plots showing the agreement between the PVP and the CVPwith their limits of agreement (LOA) and the 95% CI of the LOA according each time plot.

Legends : ARM: Alveolar Recruitment maneuver; CVP: Central Venous Pressure ; PVP: Peripheral Venous Pressure; VE: Volume expansion
