# Supplementary material for: Diagnostic accuracy of the peripheral venous pressure variation induced by an alveolar recruitment maneuver to predict fluid responsiveness during high-risk abdominal surgery
Source: BMC Anesthesiol. 2023 Jul 22;23:249. doi: 10.1186/s12871-023-02194-x (PMC10362688; doi:10.1186/s12871-023-02194-x)
Supplement: Supplementary file 4 — Supplementary Material 4 [file 12871_2023_2194_MOESM4_ESM.docx]

Supplementary Figure 1 :

ROC curves comparing the ability of variations of hemodynamic parameters induced by an ARM to predict fluid responsiveness

Legends:

∆PVP: changes in peripheral venous pressure induced by the ARM

∆CVP changes in central venous pressure induced by the ARM

∆SVI: changes in stroke volume index induced by the ARM

PPV: pulse pressure variation induced by the change of the mechanical respiratory cycle

∆MAP: changes in mean arterial pressure induced by the ARM

∆PP: changes in pulse pressure (SAP-DAP) induced by the ARM
